# Sometime

**Published:** 1875-12

**Authors:** 


### SOMETIME.

Sometime, when all life's lessons have been [learned,
And sun and stars forevermore have set,
The things which our weak judgments here [have spurned.
The things o'er which we grieved with [lashes wet
Will flash before us out of life's dark night,
As stars shine most in deeper tints of blue ;
And we shall see how all God's plans were right,
And how what seemed reproof was love most [true.
And we shall see how, while we frown and sigh
God's plans go on as best for you and me ;
How, when we called, he heeded not our cry.
Because His wisdom to the end could see.
And e'en as prudent parents disallow
Too much of sweet to craving babyhood,
So God, perhaps, is keeping from us now
Life's sweetest things because it seemeth [good.

And if, sometimes, commingled with life's wine
We find the wormwood, and rebel and shrink,
Be sure a wiser hand than yours or mine
Pours out this potion for our lips to drink.
And if some friend we love is lying low,
Where human kisses cannot reach his face,
Oh, do not blame the loving Father so,
But wear your sorrow with obedient grace !

And you shall shortly know that lengthened [breath
Is not the sweetest gift God sends his friend,
And that, sometimes, the sable pall of death
Conceals the fairest boon his love can send.
If we could push ajar the gates of life,
And stand within, and all God's workings see,
We could interpret all this doubt and strife,
And for each mystery could find a key !

But not to-day. Then be content, poor heart,
God's plans like lillies pure and white unfold;
We must not tear the close-shut leaves apart,
Time will reveal the calyxes of gold;
And if through patient toil, we reach the land
Where tired feet, with sandals loose may [rest,
When we shall clearly know and understand,
I think that we will say " God knew the best."

---

Mr. Wilson McDonald, The eminent Sculptor of 1298 Broadway, N. Y., is forming a class of a limited number of students for the Winter months, to which he will give instruction in Sculpture and Modeling· for particulars and terms apply to him by letter. Mr. McDonald is a genius, and parents having sons or daughters who show an adaptation for the noble art of sculpture, will do well to con sult him.

